# Helping Families of Infants With Persistent Crying and Sleep Problems in a Day-Clinic

**DOI:** 10.3389/fpsyt.2021.591389

**Published:** 2021-02-26

**Authors:** Binu S. K. Singh, Marina Danckaerts, Bea R. H. Van den Bergh

**Affiliations:** ^1^University of Leuven, University Psychiatric Center, Leuven, Belgium; ^2^Faculty of Medicine, University of Leuven, Leuven, Belgium; ^3^Department for Welfare, Public Health and Family, Flemish Government, Brussels, Belgium; ^4^Health Psychology Research Group, University of Leuven, Leuven, Belgium

**Keywords:** co-regulation, excessive crying, sleep problems, parent–child relation, infant, day-clinic treatment, window of tolerance, infant regulatory problems

## Abstract

Excessive crying and sleep problems affect up to 30% of infants and often coexist. Although usually benign and self-limiting, persistent crying, and sleep problems exceeding 6 months of age need attention as they may impair the mental health of the infant and its family. The source and the impact of these persistent regulatory problems is often not restricted to the infant, but extends to the parents and the parent–infant relationship. Clinical practice needs interdisciplinary and multi-method interventions focusing beyond regulatory problems of the infant but also on parental self-regulation and parent's co-regulatory responses toward the infant. Treating clinicians may encounter limitations of home-visits, outpatient, and pediatric residential settings when working with families in distress. We describe an infant mental health day-clinic treatment, drawing attention to this viable future direction. It offers a therapeutic climate based on forming a triangle of co-regulation between clinician, parent and infant to first help the parent and the infant settle down. This stress reduction restores parent–infant connectedness and parental learning and reflecting capacity. Clinicians then use established therapeutic modalities to support parental self- and co-regulatory skills which is important for the development of self-regulation in the infant. Experience with this treatment program suggests that a day-clinic setting facilitates interdisciplinary and integrative multi-method intervention, infant and parental stress reduction and integration of parental self- and co-regulatory skills in daily family life, improving overall outcomes. This perspective warrants further investigation.

## Introduction

### An Illustrative Case Report

Laura's mother sought help desperately stating: “Nobody listens, but there is something wrong with Laura and I cannot take it anymore!”. Laura, 9 months old, had had severe sleep problems since birth. She didn't sleep during the day or more than 90 min at night. When waking at night, she repeatedly needed parental support for more than 30 min before falling asleep again. Since birth, Laura had been restless, often crying, and inconsolable unless she was carried around by her mother while being comforted.

Laura's parents previously consulted many pediatricians—none of them found an organic cause. The midwife advised Laura's parents to swaddle her and the nurses' education on normal sleep and crying patterns was perceived as invalidating to the parents' concerns. Cow milk free feeding formula, a hypoallergenic diet, omeprazole, and osteopathy were also unsuccessful solutions. A behavioral approach of installing positive bedtime routines and letting Laura cry with only brief parental reassurance was ineffective.

Over time, both parents were exhausted from the 24/7 care which Laura needed and Laura's well-being and functioning became significantly impaired. Laura did not laugh, smile, or play anymore, but whined all day. The parental stress and exhaustion over time triggered maternal postnatal depression and parent-relational problems. The non-understanding attitude of the parents' social network (e.g., “You spoil her”) caused a decline in their amount of social contact and support. Both parents had little-to-no pleasant experiences with Laura since birth. Laura was perceived as a burden to the whole family, leaving little room for love and warmth.

### Persistent Sleep and Crying Problems

In the first 6 months of life, between 15 and 35% of parents report a problem with their infant's sleep and 14–29% with infant crying (1–6). These regulatory problems (RP) may occur separately, but often coexist. They are usually benign and self-limiting as transient features of normal development. However, in about 8% of infants RP persist after the age of 3 months (1, 4, 7) and have been associated with developmental and mental health problems across childhood, parental distress and poor general health, parental postnatal depression, and parent–child relationship problems with increased risk of abusive caregiver responses (1–4, 7–13). If no underlying organic cause or other infant mental health disorders are found, persistent RP exceeding the age of 6 months that impair the functioning of the infant, the family or both, are considered to be disorders which require treatment (3, 4, 7).

### Current Treatment Approaches

Persistent RP are often managed in primary care with reassurance and education of the parents about normal sleep, eating and crying patterns and/or behavioral strategies (14, 15), and/or offered the same treatment as infant colic (a self-limited phenomenon of excessive crying during the first 2–3 months of life discussed in pediatric handbooks), including dietary alterations, probiotics prescriptions and pharmacological treatment (5, 6, 16).

When primary care doesn't resolve RP, parents often turn to complementary approaches such as swaddling, herbal remedies, acupuncture, manipulative therapies, and reflexology. All of these treatment methods have poor evidence (5, 6, 16).

These linear approaches have significant limits because only in a minority of the infants an underlying organic cause is found and evidence from diverse fields of investigation of RP demonstrates that it is a complex problem. There are multiple dynamically interacting and co-evolving factors with the compromised parent–infant relationship being a main moderator of infant problem behavior and outcome: prenatal and perinatal stress factors; infant temperament and neurodevelopmental problems with self-regulatory, dietary, and gastrointestinal factors; parent's perception and tolerance to their infant's RP; and parental psychosocial risks such as parental anxiety and depression, (transgenerational) trauma, family dysfunction, and socioeconomic risks (3–7, 16–20).

Infant mental health care addresses RP by focusing on parental co-regulation within the parent–infant relationship which is necessary for the development of self-regulation in the infant. Co-regulation is a parent–infant interaction in which parents are sensitive for the signals of their infant experiencing stress and/or overwhelming emotional arousal and respond by calming their infant with soothing voice tone, warm physical contact and meeting the infant's physical and emotional needs. Repeated cycles of emotional upset, followed by relaxation after the caretakers calming intervention, provide basis for a secure attachment relationship while setting a foundation for the infant's developing self-regulation. Over time, the infant internalizes the expectation of a soothing response through which they increasingly meet their own needs and manage their own behavioral and emotional responses. Thus, according to this Mutual Regulation Model by Tronick, infants learn self-regulation skills by experiencing co-regulation in caregiving (7, 20–41).

Clinical practice needs to focus beyond RP of the infant but also on parental self-regulation and parent's co-regulatory responses toward the infant. Many effective therapeutic modalities have been developed and researched such as parent infant psychotherapy, mentalization based therapy and video feedback on parent–infant interaction. They promote parental sensitivity, meaning-making and responding to the signals of the infant; parent–infant attunement; parents' self-reflection, and awareness of how their distress, traumatic memories, and/or their own upbringing may influence their parenting (7, 20, 23, 28, 29, 37, 39–42). Treatments focused on mother–infant dyads affected by postnatal depression show promising effects on infant regulation (39, 42).

The established interventions are implemented by clinicians trained in the specific treatment modality and offered in outpatient settings, including home visiting, dyadic and group based intervention. However, the treating clinician may be challenged in how to effectively and immediately help exhausted parents who may have difficulties to engage and to transfer insights and skills from therapy sessions to daily life, compromising overall outcomes. Meanwhile, exhausted parents resort to hospital emergency departments demanding a pediatric admission.

## Approaching a Family in Distress

**Figure 1:**
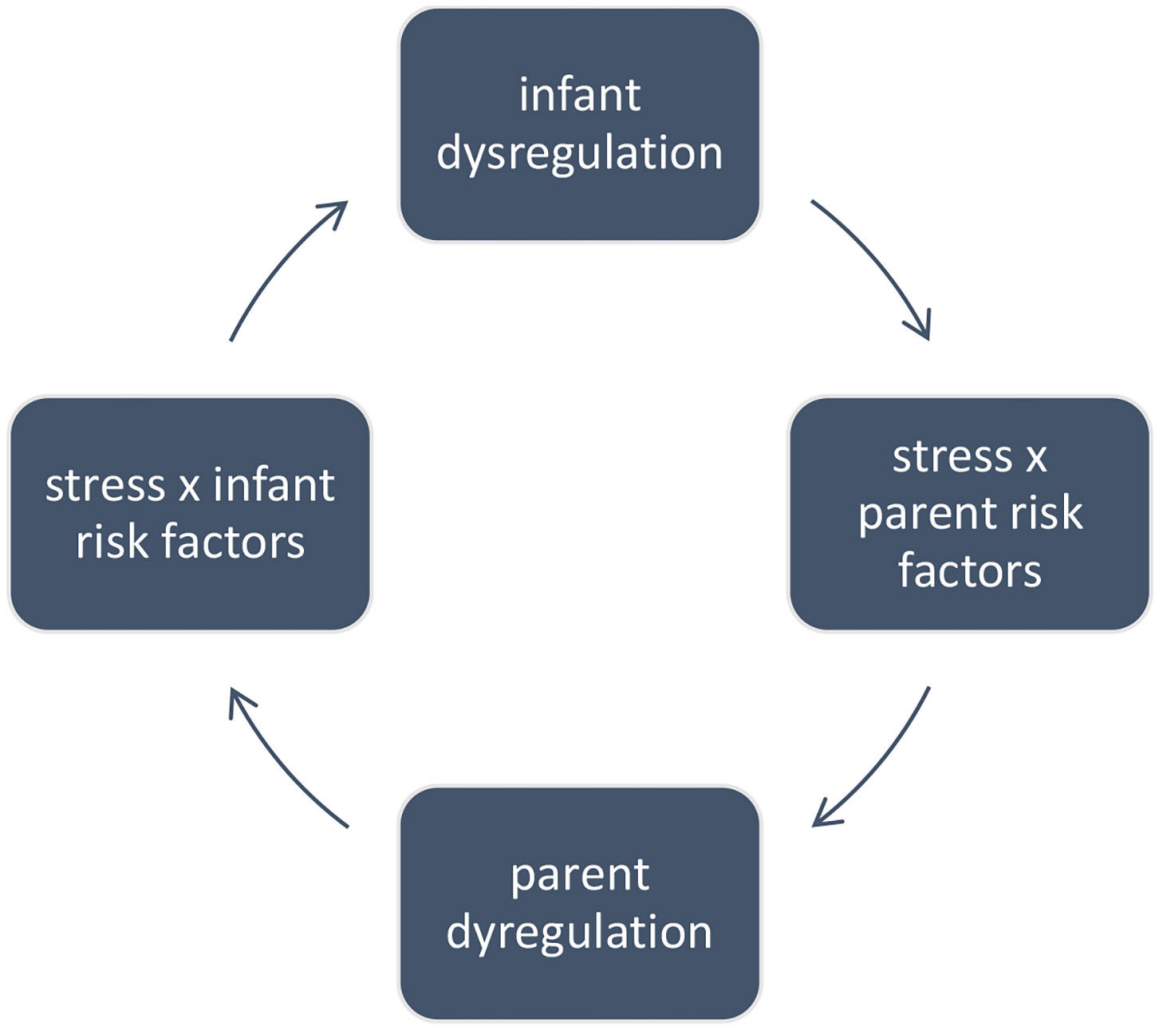
The mutual dysregulation spiral mechanism.

Infants with RP have both temperamental and physiological regulation difficulties and may be in a physical state that makes it difficult to be soothed by techniques which are usually effective (3, 7, 13, 17, 18, 20, 33, 36). This lack of responsiveness to usual care elicits acute stress reaction causing cumulative infant and parental fatigue. Parents with own vulnerabilities and lack of resources and social support are at risk to lose their own self-regulated state (7). Highly stressed and exhausted themselves, parents have no access to their own intuitive and co-regulatory skills in dealing with their infant's dysregulated behavior. As a result, a mutual parent–infant dysregulation spiraling mechanism (Figure 1) may start creating high levels of stress for both parties.

The “window of tolerance,” a term coined by Dan Siegel, is useful in motivating why stress reduction takes precedence in treatment. The window of tolerance describes the zone of arousal in which the autonomic nervous system is performing well and the brain can effectively process stimuli. In this zone, a person is likely to be able to reflect, think rationally, make decisions calmly and be socially engaged without feeling either overwhelmed or withdrawn. Extreme stress stimulates the autonomic nervous system, resulting in sympathetic hyper-arousal and parasympathetic hypo-arousal states accompanying animal defense survival responses such as fight, flight, and freeze. In either of these states, the prefrontal cortex region of the brain shuts down, and a person is said to be outside of the window of tolerance, functioning in survival mode (43). This explains why exhausted and dysregulated parents may not benefit from therapy, since the latter requires higher cognitive skills, and why parent–infant connectedness is more difficult to restore when families are in distress.

Parents need support in returning to their window of tolerance to increase their sense of calmness and connectedness as well as their learning and reflecting capacity. Then they will have the ability to deal with stress in more adaptive ways, think rationally and calmly connect with their infant and help regulate its behavioral and physiological state. An inpatient setting might facilitate providing a co-regulating space which fosters infant and parental regulated state. This might widen their window of tolerance, prevent dysregulation and at the same time amplify the existing arsenal of therapeutic approaches, making the clinician's efforts more effective.

Very little is documented or investigated on offering inpatient treatment. Wittberger describes a positive effect of an inpatient treatment with an overnight stay during 2–6 days on stress experienced by parents with excessively crying infant (44). While parents gain confidence and develop coping strategies, stress on the parents increases again upon hospital discharge.

## Treatment in a Semi-residential Infant Mental Health Setting

We describe a day-clinic treatment at the University Psychiatric Center of KU Leuven-Department Child-Psychiatry embedded in the University Hospital. It was developed between 2016 and 2020 based on literature, clinical experience, and feedback of participating families and clinical experts.

This day-clinic serves weekly 12 families of infants 3–30 months of age (demograhic data Table 1) and employs 10 clinicians for 6 full-time equivalent. It offers a multi-disciplinary and integrative multi-method approach aiming to target parental distress and ability for co-regulation of infant distress (manifested as RP).

**Table 1:** Demographics of admitted families of infants with persistent sleep and/or crying problems between 2016 and 2020.

**Demographic Data 2016–2020**	**Families** ***N*** **=** **59**	
	**No**.	****%****
**AGE OF INFANT AT ADMISSION**		
3–12 months	38	64.4
13–24 months	11	18.6
25–30 months	10	16.9
**MAIN PROBLEM OF INFANT**		
Persistent sleep problem	25	42.4
Persistent crying problem	5	8.4
Persistent sleep and crying problem	29	49.2
**FIRST CHILD OF MOTHER**		
Yes	27	45.8
No	32	54.2
**HIGHEST EDUCATIONAL LEVEL OF THE MOTHER**		
No high school	1	1.7
High school	15	25.4
Undergraduate level (associate, bachelor)	28	47.5
Graduate level (master, PhD)	9	15.3
Unknown	6	10.2

Parents apply for admission; no specific pre-assessment is needed. An interview of the parents with a simultaneous observation of the infant by a child-psychiatrist then follows to evaluate the indication for admission. Families who are caught in the mutual dysregulation spiral mechanism and for whom high levels of stress interfere with outpatient care, are admitted to the program. Families come 2 days every week, e.g. during day program for parent–infant dyads and evening program for parents. Full diagnostics and treatment is offered in collaboration with adult-psychiatry, pediatrics and Center for Infant Developmental Disorders, all located at the same hospital. Between 2016 and 2019 the admission duration varied from 3 to 37 weeks with an average of 19 weeks. The nationally funded leave system allows working parents to participate. Most of the costs are subsidized by the nationally funded mental health care system; the remaining cost for the families is 12 euros a week, if they don't have additional personal health insurance.

### Phases in Treatment: Diagnostics, Treatment, Discharge, and Aftercare

The first 6 weeks are mainly focused on diagnostics to determine a treatment plan tailored to the family's needs; nevertheless families benefit from the beginning of the therapeutic approach of diagnostics and offered group treatment. A basic diagnostic consists of interviews of parents about complaints history, developmental history, and somatic health of the infant, family history (including the childhood experiences of the parents, couple relationship, parenting development, well-being of parents and siblings, social support and financial status of the family, genetic predisposition of diseases); interview of the concerning day care; multi-disciplinary observation of the infant, parent–infant relationship and parents and scoring of DC: 0–5 observation questionnaires (3). When necessary an additional (neuro)pediatric and developmental assessment (of cognitive, verbal, and non-verbal communication and motoric skills) of the infant and mental health assessment of the parent possibly suffering from psychopathology are carried out.

Child-psychiatrist discusses the diagnosis and treatment plan with the parents for mutuality and collaboration. Progress is evaluated and treatment is refined every 6–8 weeks together with the parents. Once the mutual parent–infant regulation is established (manifested as diminishing RP in the infant, ameliorated parent–child relationship and improved well-being of the family) a discharge is prepared. If necessary, aftercare in outpatient care is started during the admission; this ensures that parents engage. Six to eight weeks after discharge families are offered follow-up with the child-psychiatrist.

### Day Treatment Program for Parent–Infant Dyad

We run two groups with infants 3–18 months of age and one group with infants 18–30 months of age, as the developmental needs are different in these age groups.

From 9 to 16 h a group of four parent–infant dyads and three clinicians meet weekly in a homey, apartment space with four bedrooms. The group moves through a fixed structure of therapeutic interventions embedded in daily life routines and with room for individual attuned support: music and dance therapy, Greenspan floortime, mindful parenting, baby massage, Sherborne developmental movement, mentalization based therapy, parent–infant psychotherapy, pre- and perinatal baby therapy alternate with eating, nursing, playing, resting, putting infants to sleep, having short conversations with peers or clinicians and going for a walk in nature (40, 45–54).

The core element is providing a co-regulating and “holding” therapeutic climate which fosters infant and parental regulated state by the installment of the “chain and triangle of co-regulation”.

Clinicians, who are themselves adequately self-regulated, form a triangle with the parent–infant dyad to first downregulate their stress and negative affect. They use their own (social) connectedness and create a safe supporting surrounding emphasizing their own physical and emotional availability and the person-to-person attunement to help the parent and the infant settle down. Releasing stress enables the parent and the infant to be more emotionally available to connect, communicate, and interact with each other.

Clinicians then form a chain with the downregulated parent–infant dyad to support parents co-regulate their infant. Clinicians model self- and co-regulatory skills and practice them together with parents. When their infant shows dysregulated behavior clinicians help parents to remain calm, be sensitive to their infant's cue and respond with new soothing behavior.

In this way, the chain and triangle of co-regulation open the door for infants and their families to shift and settle from the mutual dysregulation to mutual regulation. Concurrently, the parents benefit from peer-support during the process.

### Evening Treatment Program for Parents

From 16 to 20.30 h one evening every week, the parents join (alone or as couple) a therapeutic program embedded in an informal meeting space, where they may have a peaceful meal and some leisure time with their spouse and peers. The therapeutic program offers three sessions: a semi-structured group intervention offering parental education and enhancing peer support, a downregulating group sessions to experience several ways of self-regulation (such as mindfulness, yoga, walking in nature, breathing exercises, etc.), and an individual therapeutic session tailored to the family's needs (offering parenting advice, parent–infant psychotherapy, video feedback, mentalization based therapy, trauma therapy, couple therapy, session promoting parental self-care and social support to enhance parental resilience, etc.). Parents appreciate the combination of these evenings with the day treatment program and being at home in between: discussing problems encountered during day treatment or at home with each other, peers and clinicians, and practicing new insights together with clinicians during day treatment and subsequently at home themselves, helps parents to come to sustainable changes.

## Discussion

Our clinical experience suggests that a day-clinic setting facilitates an interdisciplinary and integrative multi-method intervention, infant and parental stress reduction and integration of parental self- and co-regulatory skills in daily family life, making the clinician's efforts more effective when treating distressed families of infants with severe and persistent RP. Compared to home visiting or outpatient care parents find it helpful shutting out the daily stress factors for one day, just to experience what it means to them and their baby. This contrast reveals the changes to be made at home and motivates parents. Parents appreciate having a full day, week after week, with many opportunities to observe and learn together with clinicians in many different ways. Due to collaboration with adult-psychiatry and pediatrics, organizing after-care during admission at the day clinic and follow-up after discharge, parents seem to experience less fragmentation and discontinuity in care. Peer support helps parents to normalize their experiences, counter feelings of guilt/failure, destigmatize and break through the social isolation. Observing and listening to other parents' experiences may shed light more spontaneously on issues parents had not thought of themselves or are not willing to confront. Parents learn from each other: “seniors” reassure “juniors” in their doubts and struggles and “juniors” show “seniors” how far they have progressed; live testimonials from discharged families offer hope.

This day-clinic treatment intends to be complimentary and collaborative with existing perinatal and infant mental health care.

Mother-baby units primarily focus on mothers' psychiatric disorders, while this treatment program primarily focuses on the infant RP and needs the parent to mediate the treatment for the infant and therefore also supports the parents. In case of severe parental psychopathology the parent is referred to adult psychiatric care, such as mother-baby units. If necessary, the infant may be treated parallel or subsequently at the day-clinic.

Compared to residential care, a part-time admission at day-clinic allows families to integrate what they have learned in their home environment and continue their normal life, e.g., work, social contacts, care for siblings, etc. This might facilitate transfer, lower the risk of relapse upon discharge and make the admission practically and financially feasible. But for infants living in unsafe home environment, other care is needed, such as facilities ensuring safety of the infant, mother-baby units, and family units.

The treatment we described is an intensive and costly approach, challenging parents work-life balance, challenging health services to form a bridge between services for adults' and infants' well-being and mental health, and challenging focus of subsidy by public health policy and modalities of payment by health insurance companies. The duration of the treatment is long, but it targets severe and persistent infant RP requiring intensive treatment, if only to manage the secondary impact. However, it is a challenge and important to investigate the difference between spontaneous recovery and the effect of the treatment.

Clearly, more research is needed to study the short and long-term effectiveness of the intervention we described.

## Laura's Illustrative Case Report: Day-clinic Treatment With Co-regulation At the Core

Taking the first step toward co-regulating the distress in Laura's family, her mothers' concerns were validated and her father was involved from the beginning. In treatment, Laura's parents slowed down and together with peers and clinicians observed and experienced the distinct ways in which self-regulation affected themselves and their infant. They reduced their daily stress due to parental leave of Laura's father, household help, support of grandparents, and Laura's successful start at day care. These changes created more quality time for the parents to spend with Laura and alone as marital partners, fostering the family's resilience. Simultaneously, this improved their ability to benefit from the psychiatric treatment for the mother's depression, parenting advice with video feedback and couple therapy. Laura's parents were more calm and emotionally available, learned to interpret Laura's cues more quickly and were able to successfully soothe her. They shared more pleasurable interaction with their daughter. Gradually, Laura's crying and whining diminished and she appeared to be happier and more relaxed. Laura began sleeping during the day as the parents improved their perception of self-efficacy in caring for Laura. Now under these circumstances previously failed behavioral strategies such as gradual extinction and positive bedtime routines effectively helped Laura to sleep for longer periods at night.

During these 5 months of treatment Laura became a gift for the wellbeing of her whole family instead of a burden, when her parents realized that helping Laura started by helping themselves.

## Data Availability Statement

The original contributions presented in the study are included in the article/supplementary material, further inquiries can be directed to the corresponding author/s.

## Ethics Statement

Written informed consent was obtained from the individual(s), and minor(s)' legal guardian/next of kin, for the publication of any potentially identifiable images or data included in this article.

## Author Contributions

BS, drafting the manuscript. BV and MD, critical revision of the article. BS, BV, and MD, final approval of the version to be published. All authors contributed to the article and approved the submitted version.

## Conflict of Interest

The authors declare that the research was conducted in the absence of any commercial or financial relationships that could be construed as a potential conflict of interest.
